# Antimicrobial, Anti‐Biofilm Activity and Antioxidants of Phenolic Compounds Isolated From *Hypericum perforatum* on Periodontal Pathogenic Oral Bacteria

**DOI:** 10.1002/fsn3.70336

**Published:** 2025-05-23

**Authors:** Siba Mouid Al‐haliem, Muthanna Jasim Mohammed, Mohammad Ali Hesarinejad, Tarek Gamal Abedelmaksoud

**Affiliations:** ^1^ Department of Dental Basic Sciences, College of Dentistry University of Mosul Mosul Iraq; ^2^ Department of Biology, College of Education for Pure Sciences University of Mosul Mosul Iraq; ^3^ Department of Food Sensory and Cognitive Science Research Institute of Food Science and Technology (RIFST) Mashhad Iran; ^4^ Food Science Department, Faculty of Agriculture Cairo University Giza Egypt

**Keywords:** anti‐biofilm, antimicrobial, antioxidants, *Hypericum perforatum*, periodontal, phenolic compounds

## Abstract

Wild plants are a rich source of phenolic compounds with antimicrobial and antioxidant properties. This study extracted and analyzed *Hypericum perforatum* leaf fractions for their phenolic profile, antioxidant capacity, and activity against antibiotic‐resistant oral bacteria. Phenolic compounds were identified using column chromatography, TLC, and HPLC. Four different fractions, two from ethyl acetate extraction and two from ethanolic extraction, were obtained and evaluated further. The antimicrobial activity of each fraction was assessed against two Gram‐negative bacteria that cause gum disease, *Morococcus cerebrosus* and *Eikenella corrodens* , using the disc‐diffusion assay. Fraction I exhibited the most potent antimicrobial activity against *E. corrodens* and *M. cerebrosus* , with 20–25 mm inhibition zones at lower concentrations. Fraction II showed limited activity, primarily at 100 and 200 μg/mL, while Fractions III and IV had moderate effects, with some concentrations effective against *E. corrodens* . Regarding biofilm formation, Fraction I showed the most significant reduction Fraction I showed the most significant reduction in biofilm formation for both *E. corrodens* and *M. cerebrosus* at 50 and 200 μg/mL concentrations. Fraction II demonstrated variable effects, with an increase in biofilm formation at 200 μg/mL for *E. corrodens* . At the same time, Fractions III and IV had moderate reductions in biofilm formation across most concentrations. The antioxidant activity of *H. perforatum* fractions, assessed via DPPH, surpassed Vitamin C at lower concentrations, with Fraction II showing the highest activity (83.98% at 500 ppm). These findings highlight *H. perforatum* as a promising natural source of phenolics with potential applications in managing periodontal infections.

## Graphical abstract

The study on *Hypericum perforatum* revealed its leaf fractions possess potent antimicrobial and antioxidant properties against periodontal bacteria, specifically *Eikenella corrodens* and *Morococcus cerebrosus* . Fraction I exhibited the highest antimicrobial activity, with significant inhibition zones, and effectively reduced biofilm formation. Additionally, the antioxidant capacity of the fractions surpassed that of Vitamin C at lower concentrations, highlighting the potential of *H. perforatum* as a natural therapeutic agent for managing periodontal infections.

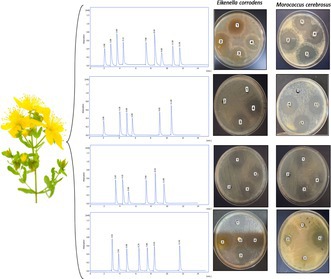

## 1 Introduction

*Hypericum perforatum* , commonly known as St. John's Wort, is a perennial herbaceous plant belonging to the *Hypericaceae* family; this genus includes hundreds of species; its original homeland is the Middle East. This genus is spread in various world regions, except for tropical depressions, deserts, and polar regions. The *Hypericaceae* family consists of more than 450 species, but only sixteen species are found in Iraq (Kwiecień et al. 2021; Hoshyar and Kadhim 2013) and grow to a height of between 20 and 50 cm. Its leaves are characterized by containing many glands filled with transparent aromatic oil. These leaves are opposite, green in color, and either elongated or oval‐shaped with a smooth surface. The stem is cylindrical, from which numerous leafy branches emerge. These branches end with yellow flowers (Kaplan et al. 2023).

The most commonly used part of *H. perforatum* is the leaves; *H. perforatum* leaves contain compounds that exhibit significant antibacterial and anti‐inflammatory effects against bacteria associated with periodontitis (Bagheri et al. 2022). *Hypericum perforatum* contains various chemical compounds, including volatile oils, flavonoids, anthraquinone derivatives, phenols, tannins, xanthones, and other diverse compounds. It also contains other compounds: phloroglucinols such as hyperforin, hypericin, and pseudo hypericin, and flavonoids like quercetin, quercitrin, rutin, and hyperoxide. However, the most important compounds in this plant are phenolic compounds of all kinds, as they are the target of many studies due to their significant effectiveness against many stubborn diseases (Rychlewski et al. 2023; Yi et al. 2021).

In dentistry, aggressive periodontitis (AgP) is a severe periodontal disease characterized by rapid attachment loss and bone destruction, often leading to tooth loss if untreated. It is primarily driven by the accumulation of dental biofilm, which triggers disproportionate inflammatory responses from the host, resulting in significant tissue damage (Łasica et al. 2024). Biofilm formation is one of the important challenges in combating oral diseases, which are complex structures consisting of microorganisms permanently attached to oral surfaces and embedded within an extracellular polymeric matrix (EPS). These structures alter the properties of microorganisms compared to those in their free‐floating state, granting those 100–1000 times higher resistance to antibiotics and antimicrobials (Wade 2021; Donlan and Costerton 2002). Antimicrobial agents are used to prevent and treat oral infections by reducing the number of bacteria present in the mouth. However, there is an increasing need to discover new materials due to bacteria's rising primary and secondary resistance to synthetic antibiotics and disinfectants (Badr et al. 2023; Łasica et al. 2024). Given the growing inefficacy of synthetically produced agents, plant‐derived compounds have gained interest in developing therapies to control oral biofilms (Karygianni et al. 2019).

The most important bacteria that cause gum disease are *Morococcus cerebrosus* , which belongs to the family *Neisseriaceae*, contributes to the calcification of dental plaque, and was identified and associated with brain abscesses (Veras et al. 2023). *Morococcus cerebrosus* is a gram‐negative coccus and oxidase‐positive, forming clusters resembling berries with a diameter of less than one micrometer. This organism is non‐motile, does not form endospores, and is aerobic. It plays a role in the complex microbial interactions contributing to periodontal inflammation, mainly through its relationship with other pathogens such as *Porphyromonas gingivalis* (Mahdi et al. 2024). As for bacteria, *Eikenella corrodens* , a distinct species within the genus *Eikenella*, belongs to the HACEK group of microorganisms (Matheus et al. 2023). It is a small, gram‐negative, facultative anaerobic rod that is non‐motile and difficult to motivate. It can cause brain and liver abscesses (Stormo et al. 2020). Biofilm detachment is critical for the spread of various pathogens, and the presence of *M. cerebrosus* and *E. corrodens* may promote biofilm maturation and periodontal disease transmission and thus make it resistant to antibiotics (Karim et al. 2013).

The accumulation of free radicals initiates various diseases. Neutralizing free radicals is made possible through antioxidants. The mechanistic definitions of antioxidants generally focus on their ability to act as hydrogen or electron donors (Martemucci et al. 2022). Research continues to discover natural sources that can serve as alternatives to synthetic antioxidants. Natural plant‐derived antioxidants, mainly phenolic compounds, are featured. They are safe, relatively low‐cost, and readily available. Compared to synthetic antioxidants (Guler and Okmen 2024; Faris et al. 2024), they function as single and triple oxygen quenchers, free radical scavengers, peroxide decomposers, enzyme inhibitors, and synergists (Usin and Daramola 2022). This study specifically aims to investigate the antimicrobial activities against various gum disease pathogens and the antioxidant activity of natural phenolic compounds separated from the leaves of *H. perforatum* .

## 2 Materials and Methods

### 2.1 Preparation of *H. perforatum* L. Extract by Soxhlet Apparatus

Leaves of *H. perforatum* L. (St. John's wort) were collected in June–July 2024, in North Mosul city (24.0000″ N and 43°70 48.0036″ E) during the flowering season. The plant was washed with water several times to remove dust, soil, and dirt. The leaves were manually separated, and the samples of the leaves were left to shade‐dry at room temperature for 10 days. After 10 days, the dried samples were ground using an electric grinder with a blade (food‐grade stainless steel) (Huangcheng, China). Homogeneous powder was obtained in a fine powder. Then, extracts from the plant leaves were prepared using the Soxhlet apparatus (1000 mL LabTech—American/Chinese). Firstly, 100 g of crushed leaf powder/paste was processed/extracted using the Soxhlet apparatus successively with different conventional organic solvents (1000 mL), namely hexane, ethyl acetate, and ethanol, to increase polarity. The hexane extract was excluded as it was used as a non‐polar solvent in defeat. The extraction process was carried out at a constant temperature of 60°C for 72 h in a water bath and was repeated three times for each solvent. A rotary evaporator was used to evaporate the crude plant extract. Once the organic solvent evaporated, the extract was filtered with a Whatman No. 1 filter to remove undesired materials. The two extracts were weighed and stored in sterile, dark, airtight containers for further analysis, as phenolics are prone to photodegradation (Al‐Assaf et al. 2024).

### 2.2 Isolation and Fractions of *H. perforatum* Extract by Column

Column chromatography (CC) was employed to isolate and fractionate the ethyl acetate and ethanol extracts from the leaves of *H. perforatum* L. The column was packed using 250 g of silica gel (60–120 mesh) as the stationary phase, prepared by the wet packing method with hexane as the dispersing solvent. A slurry of silica gel in hexane was carefully poured into the column to ensure uniform packing. The plant extracts, pre‐mixed thoroughly with a small quantity of silica gel, were gently layered on the packed column. Elution was performed using a gradient of solvent mixtures, including hexane, ethyl acetate, and ethanol, as the mobile phases. The chromatography process was initiated by opening the column's stopcock to allow the mobile phase to flow through, with additional solvent continuously added to prevent the stationary phase from drying out. Following elution, the collected fractions were concentrated and screened using thin‐layer chromatography (TLC) to identify those containing phenolic compounds. Only fractions eluted with ethyl acetate and ethanol were subjected to further TLC analysis, as the hexane fraction remained difficult to separate and retained a crude appearance (Sidoryk et al. 2018).

### 2.3 HPLC System and Conditions

The high‐performance liquid chromatography (HPLC) method was developed and optimized using comprehensive validation parameters to ensure accurate and reproducible results. The elution order of phenolic compound standards was established by analyzing separate solutions containing 20 μg/mL of individual analytes. The analysis was performed using a SYKAM HPLC system (Germany) with a C18‐ODS column (250 × 4.6 mm, 5 μm), selected for its precision and reliability. Sample injections of 100 μL were introduced into the system. The mobile phase consisted of the following solvents: Solvent A: 95% acetonitrile +0.01% trifluoroacetic acid; Solvent B: 5% acetonitrile +0.01% trifluoroacetic acid. The gradient program was meticulously designed for optimal separation, progressing as follows: 10% A from 0 to 5 min; 25% A from 5 to 7 min; 40% A from 7 to 13 min; returning to initial conditions. The flow rate was maintained at 1 mL/min throughout the analysis. Phenolic compounds were detected using a UV–Visible detector set at a wavelength of 278 nm, ideal for their characteristic absorption spectra. Matching the retention times of peaks in the samples to those of the corresponding standards confirmed the identity of individual phenolic compounds. Twelve standard phenolic compounds were analyzed, as detailed in Table 1.

**TABLE 1:** Standards of phenolic compounds and their retention time.

Standards	Retention time (min)	Concentration (ppm)	Area (mAU.s)
Rutin	2.05	5	1203.65
Qurcetin	3.01	5	1598.80
Kaempferol	3.81	5	1652.65
Ferulic acid	4.23	5	1425.49
Caffeic acid	5.08	5	1741.05
Apigenin	5.85	5	1354.19
Gallic acid	6.21	5	1236.62
Luteolin	7.85	5	1320.25
P‐coumaric acid	8.50	5	1456.98
Chlorogenic acid	9.80	5	1230.65
Syringeic acid	11.05	5	1524.98
Sinapic acid	11.98	5	1321.02

### 2.4 Sample Collection

Twenty samples, 12 males and 8 females, were collected from patients with aggressive periodontitis, depending on clinical examination by a specialist dentist at the Dental Specialized Dental Centre/Officers in Mosul. Absorbent paper points (size 30) were inserted into the base of the pocket for 30–60 s and then transferred to tubes containing a brain‐heart infusion medium. The patient's average age is 18–45 years for both sexes (Al‐Hamdoni and Al‐Rawi 2020). Tubes containing isolated samples were inoculated on blood agar media and incubated aerobically and anaerobically at 37°C for 2–5 days, then identified based on colony morphology, biochemical tests, and molecular methods. Each isolate's 16S rRNA gene sequence was determined and compared with the National Center for Biotechnology Information (NCBI) sequence.

### 2.5 Investigation of the Ability of Bacteria to Form Biofilm

Investigation of the ability under study to form biofilms. Using the Tube Method (TM), A loopful of pathogenic bacteria used in the study was placed in 10 mL of Trypticase soy broth medium containing 1% glucose in glass containers, each separately. The bacterial cultures were incubated for 24–48 h. After the incubation period, the tubes were poured out and rinsed with a phosphate‐buffered saline solution (with a pH of 7.3) and then dried. Then, crystal violet dye was added to them at a concentration of 1%, and the dye was left for 30 min. The excess dye was removed and washed with sterile distilled water several times. The containers were placed upside down and left to dry at laboratory temperature. Biofilm formation was deemed positive if a thick film was visibly present on the tube's wall and bottom (Ibrahim 2016).

### 2.6 Investigation of the Inhibitory Activity Fractions

It was assessed by the diffusion method; one or two isolated colonies of bacteria from the original culture were chosen, and they were then placed into a test tube with four milliliters of normal saline to create a bacterial suspension of moderate turbidity. This procedure was compared to the standard turbidity solution that was prepared. This comes out to about 1.5 × 108 CFU/mL. A part of the bacterial suspension was carefully and uniformly placed using a sterile cotton swab over Mueller‐Hinton agar medium, and it was then incubated for 10 min. Using a sterile cork punch with a diameter of 6 mm, holes were made on the surface of the plate, and 10 μL of each concentration of the plant extracts, separate phenolic compounds, and synthetic nanoparticles were placed in each hole. The plate was incubated at 37°C for 24 h. Then, the diameters of the growth inhibition zones surrounding each hole were measured in mm using a graduated ruler. The results were then recorded and compared with the effects of antibiotics (Balouir et al. 2016).

### 2.7 Investigation of the Ability of Fractions to Prevent the Formation of Biofilms

The effect of phenolic compounds isolated from the *H. perforatum* plant at different concentrations towards the formation of biofilms by isolated pathogenic bacteria was studied using micro‐well plates (M.T.P.). In 96 wells, 20 μL of the bacterial suspension diluted with pathogenic bacteria was transferred to each well in 200 μL of nutrient broth medium. 10 μL of each concentration of phenolic compounds was added to the wells containing the bacterial isolates, with control wells containing only the nutrient medium and other wells containing the nutrient medium inoculated with bacteria. Then, the M.T.P. plates were incubated for 24 h at 37°C. After the incubation period, the wells' contents were removed using a fine pipette. The wells were washed 3 times with phosphate buffer solution, and then 95% ethanolic alcohol was used to fix the cells attached to the walls of the wells. The plates were left for 10 min; the alcohol was removed and left to dry in the air. After that, the wells were stained with 1% crystal violet stain by adding 100 μL to each well. The staining process was stabilized for 15 min; then, the wells were washed with distilled water from the cannabis plant. The heart in the formation of biofilms was observed by observing the transparent color gradient with crystal violet dye. The dye attached to the walls of the holes was then extracted by adding 100 μL of 33% glacial acetic acid to each hole. Then, the optical density of the biofilms was measured using a spectrophotometer at a wavelength of 630 nm. (Mathur et al. 2006).

### 2.8 DPPH Assay for Antioxidant Activity

The DPPH assay was conducted using a modified method to evaluate the antioxidant activity of the obtained fractions. The test utilized DPPH (2,2‐diphenyl‐1‐picrylhydrazyl), a stable free radical. Fractions were prepared at varying concentrations (10, 20, 40, 80, and 160 μg/mL) and dissolved in 1 mL of ethanol. Subsequently, 20 mg of DPPH dissolved in 100 mL of ethanol was added to each sample, ensuring a comprehensive evaluation process. The mixtures were thoroughly shaken and incubated at 37°C for 30 min in dark conditions to prevent light‐induced degradation. After incubation, the absorbance was measured at a wavelength of 517 nm (λ 517 nm). Ascorbic acid, a water‐soluble vitamin with potent antioxidant properties, was employed as the standard reference. This benchmark allowed for the comparison and validation of the results, providing a reliable measure of the antioxidant activity of the samples.

The percentage of DPPH scavenging activity (% inhibition) was calculated using Equation (1), and the antioxidant activity was further expressed as the EC50 value. The EC50 represents the sample concentration required to reduce 50% of the DPPH radicals, a critical indicator of the sample's antioxidant potency (Al‐Snafi 2016).

(1) $$\begin{array}{c} \%\text{DPPH scavenging}\\ =\left(1-\mathrm{Abs}\;\text{sample}–\mathrm{Abs}\;\text{blank}\;\mathrm{Abs}\;\text{control}–\mathrm{Abs}\;\text{blank}\right)\times 100\% \end{array}$$

## 3 Results and Discussion

### 3.1 Composition of Phenolic Compounds in *H. perforatum* Fractions

**FIGURE 1:**
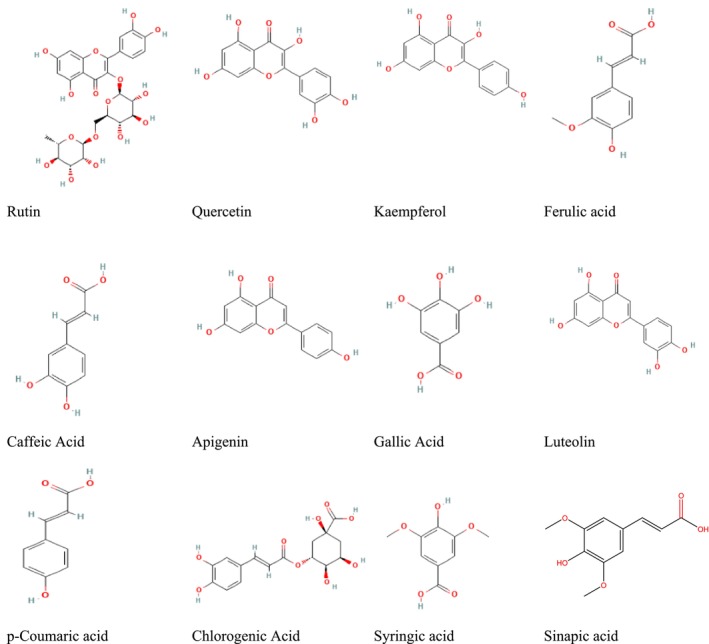
2D‐chemical structure of phenolic compounds extraction from *Hypericum perforatum*.

**FIGURE 2:**
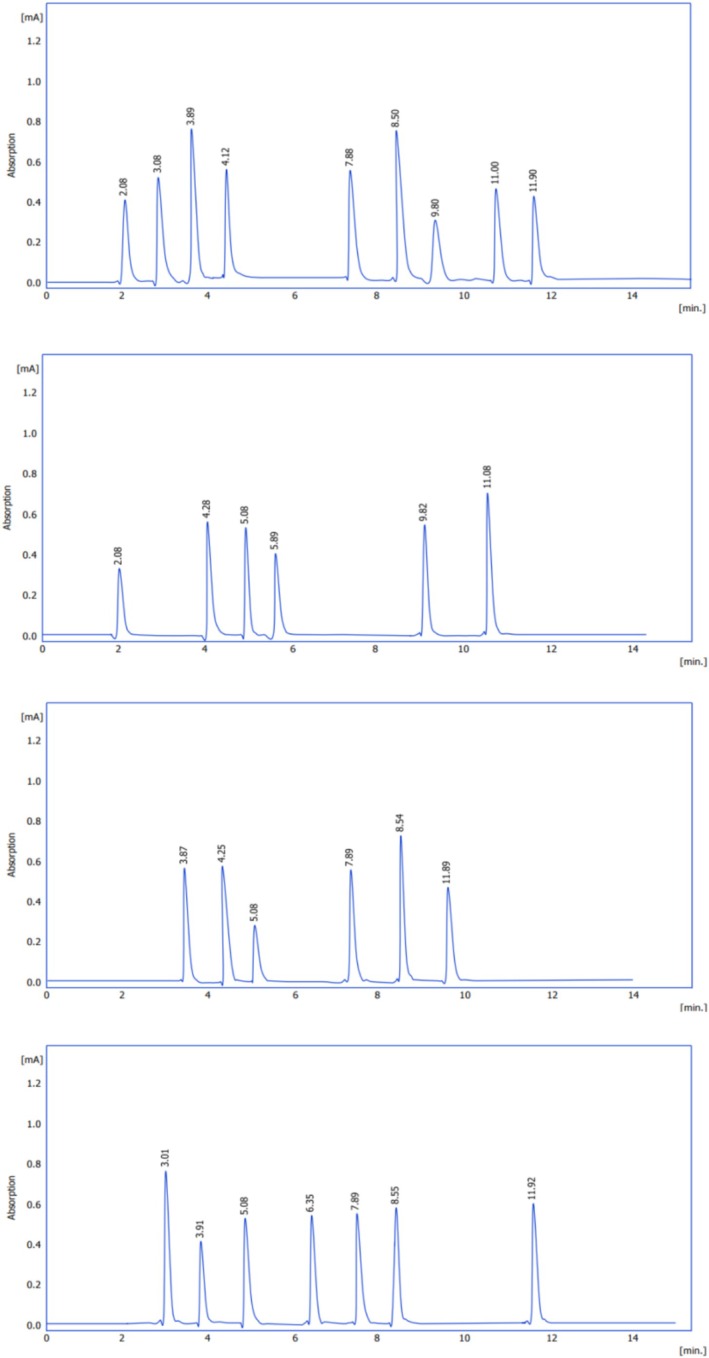
HPLC chromatogram of fraction I–IV.

Table 2 outlines the phenolic compounds identified in different fractions from ethyl acetate and ethanol extractions, comparing the number of compounds, their concentrations, and their relevance as antioxidants and antibacterials. Fraction I (ethyl acetate) contains 9 compounds, including rutin, quercetin, kaempferol, ferulic acid, luteolin, p‐coumaric acid, chlorogenic acid, syringeic acid, and sinapic acid, with concentrations ranging from 10.25 ppm (chlorogenic acid) to 23.76 ppm (kaempferol), highlighting its potential for both antioxidant and antibacterial applications, mainly due to compounds like quercetin and kaempferol. Fraction II (ethyl acetate) has 6 compounds, with concentrations from 9.96 ppm (chlorogenic acid) to 23.87 ppm (caffeic acid), containing potent antioxidants such as caffeic acid and chlorogenic acid, which are known for reducing oxidative stress and inflammation. In comparison, Fraction III (ethanol) has 6 compounds, with a range of concentrations from 8.71 ppm (caffeic acid) to 23.85 ppm (luteolin), including kaempferol, luteolin, and sinapic acid, which are significant for both their antibacterial properties and antioxidant potential. Fraction IV (ethanol) also contains 7 compounds, with concentrations varying from 12.56 ppm (kaempferol) to 20.60 ppm (quercetin), offering similar bioactive properties as Fraction I, particularly with the inclusion of quercetin and gallic acid, known for their antibacterial and antioxidant activities, as in Figures 1 and 2. Overall, the ethyl acetate fractions (I and II) tend to have higher concentrations of bioactive compounds, while the ethanol fractions (III and IV) offer a slightly different profile of phenolic compounds, all of which are valuable for therapeutic uses such as reducing oxidative stress and combating bacterial infections.

**TABLE 2:** Phenolic compounds in different fractions and their retention time.

Fractions	Number of peak	Retention time (min)	Concentration (ppm)	Identified compounds
I	1	2.05	18.78	Rutin
	2	3.01	16.37	Qurcetin
	3	3.81	23.76	Kaempferol
	4	4.23	18.28	Ferulic acid
	5	7.85	20.49	Luteolin
	6	8.50	21.31	P‐coumaric acid
	7	9.80	10.25	Chlorogenic acid
	8	11.05	11.03	Syringeic acid
	9	11.98	12.15	Sinapic acid
II	1	2.08	10.65	Rutin
	2	4.28	18.18	Ferulic acid
	3	5.08	11.92	Caffeic acid
	4	5.89	9.96	Apigenin
	5	9.82	23.87	Chlorogenic acid
	6	11.08	23.51	Syringeic acid
III	1	3.87	13.67	Kaempferol
	2	4.25	16.09	Ferulic acid
	3	5.08	8.71	Caffeic acid
	4	7.89	16.54	Luteolin
	5	8.54	23.85	P‐coumaric acid
	6	11.89	13.72	Sinapic acid
IV	1	3.01	20.60	Qurcetin
	2	3.91	12.56	Kaempferol
	3	5.08	14.31	Caffeic acid
	4	6.35	20.27	Gallic acid
	5	7.89	18.89	Luteolin
	6	8.55	16.99	P‐coumaric acid
	7	11.92	19.00	Sinapic acid

*Note:* Fractions (I and II) were identified from ethyl acetate extraction; Fractions (III and IV) were identified from ethanol extraction.

The HPLC method exhibits unique selectivity and sensitivity because it combines high‐performance liquid chromatography with strong separation ability. This technique is fast, specific, and delicate. It is one of the most efficient processes for determining metabolites and has become a key tool in the metabolic exploration of plant extracts (Alahmad et al. 2022; Altemimi et al. 2023).

*Hypericum perforatum* extracts are a rich source of several classes of plant phenolics with documented biological activity. These include antidepressant phloroglucinols (hyperforin and its derivative adhyperforin), antiviral, antibacterial, and photosensitizing naphthodianthrones (hypericin and pseudohypericin, as well as their precursors—protohypericin and protopseudohypericin), antioxidant flavonoids (mostly quercetin and kaempferol glycosides and aglycones, as well as bioflavonoids), and phenolic acids (mostly isomeric caffeoylquinic acids). For pharmaceutical product production, preparing the enriched extracts may be of interest. It has been demonstrated that it is possible to obtain extracts with high levels of phloroglucinols and naphthodianthrones by using a relatively simple procedure, which should inspire optimism about the ease of production. The antioxidant activity of *H. perforatum* extracts is well known and is to be expected due to the high content of phenolic compounds. However, several publications were focused on this topic (Orčić et al. 2011). In the *Hypericum* species studied, ten phenolic acids (gallic, protocatechuic, p‐hydroxybenzoic, caffeic, chlorogenic, syringic, p‐coumaric, ferulic, tr‐cinnamic, and coumaric acids) were determined by an HPLC gradient system using a modified method, which was described elsewhere (Öztürk et al. 2009).

The total flavonoid content of the different *H. perforatum* L. samples were measured, leading to some novel and intriguing findings. The total flavonoid content value in water and ethanol extracts varied between 598–629 mg and 635–690 mg in 100 g of dry weight. The ethanol extracts, in particular, proved to be more prosperous in phenolic compounds than the water extracts. The ethanol extracts of three different samples showed no significant differences in the qualitative but rather considerable variability in the quantitative composition of the solutions. These findings pique interest in the variability of *H. perforatum* L. The qualitative analyses of *H. perforatum* L. indicated that most polyphenols are in glycoside form, and the main flavonoid aglycone is quercetin. The other aglycones are quinic, protocatechuic, vanillic, caffeic, coumaroylquinic, and OH‐phenylpropionic acids, catechin, and epicatechin monomers and oligomers. *H. perforatum* L. contains three different chlorogenic acids: chlorogenic acid (5‐O‐caffeoylquinic acid), neochlorogenic acid (3‐O‐caffeoylquinic acid), and cryptochlorogenic acid (4‐ O‐caffeoylquinic acid), identified by their negative MS/MS fragmentation spectra (comparison with commercial chlorogenic acid and literature data) as well as by chromatographic retention times (Helmja et al. 2011). In the *H. perforatum* extract, the main flavonoid component is hyperoside (665.38 mg %) followed by isoquercitrozide (569.08 mg %), rutoside (462.82 mg %) and quercitrozide (110.22 mg %). Free flavonoid aglycones are represented by quercetin (533.03 mg %) and kaempferol (11.46 mg %) in a much lower amount. These aglycones also appear in the unhydrolyzed extracts, but in a much smaller amount. The free phenylpropane compounds are represented by gentisic acid, chlorogenic acid, and caffeic acid, whose presence was confirmed by SM and by p‐cumaric acid (9.59 mg %) and ferulic acid (5.62 mg %) (Gitea et al. 2018).

### 3.2 Antimicrobial Activities of *H. perforatum* Fractions

**FIGURE 3:**
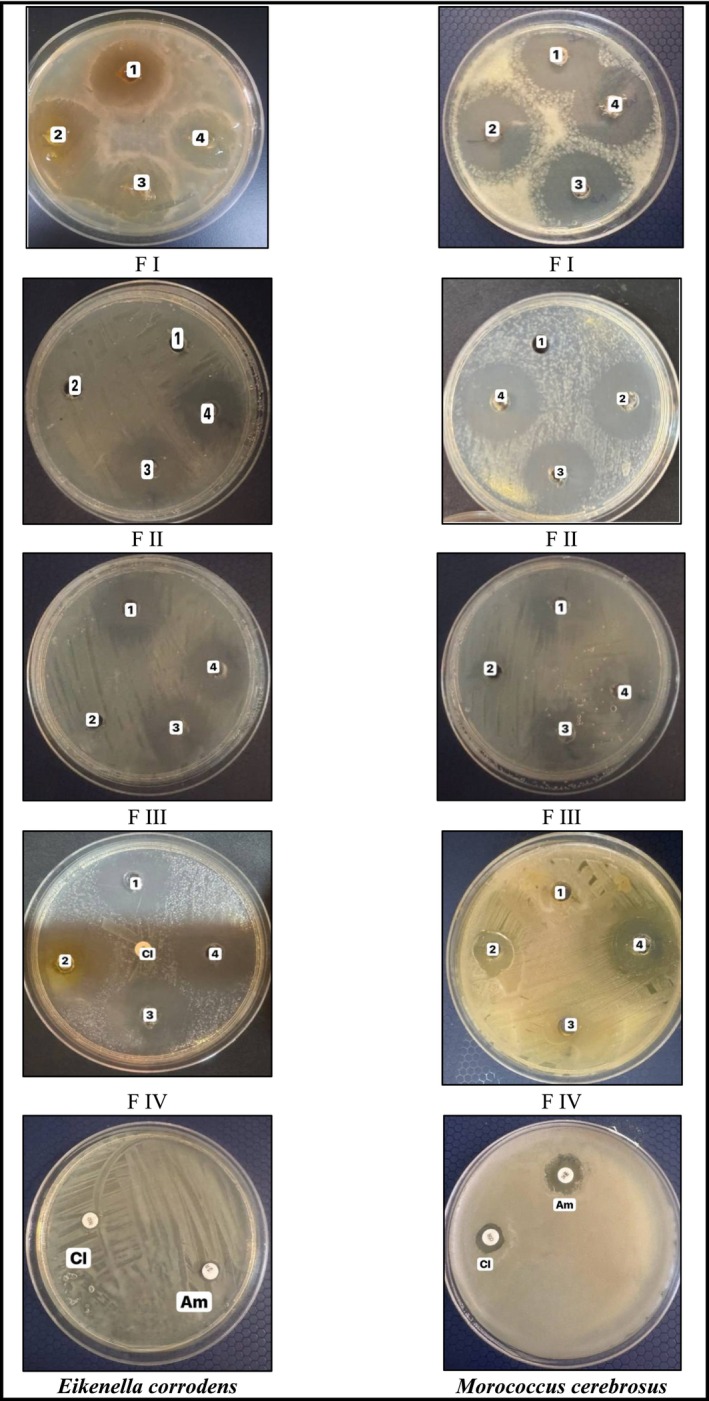
Antimicrobial activity of fraction I–IV against *Eikenella corrodens* and *Morococcus cerebrosus* .

Table 3 presents the antibacterial activity of phenolic compounds isolated from *H. perforatum* against *E. corrodens* and *M. cerebrosus* . These findings are significant as they provide insights into the potential use of natural compounds in combating bacterial infections. For fraction I, the inhibition zones varied from 20 mm to 25 mm for both bacteria at 25 μg/mL and 50 μg/mL, but dropped to 20 mm at 100 μg/mL for *E. corrodens* and *M. cerebrosus* at 200 μg/mL. Fraction II exhibited no inhibition at 25 μg/mL and 50 μg/mL, with moderate activity (19 mm at 100 μg/mL) against *E. corrodens* and a more substantial zone of 21 mm at 200 μg/mL for *M. cerebrosus* . Fraction III showed partial inhibition at 25 μg/mL and 100 μg/mL, with 19 mm and 18 mm zones, respectively, but no activity at 50 μg/mL. Fraction IV demonstrated weak inhibition at 25 μg/mL (13 mm) but improved at 50 μg/mL (18 mm) and showed good inhibition at 100 μg/mL (23 mm) against *E. corrodens* , while having no effect against *M. cerebrosus* at 25 μg/mL, with the highest zone of 22 mm at 200 μg/mL. When compared to antibiotics, ampicillin and cephalosporin (cephriaxim) had minimal effect, with inhibition zones of 0 mm for both bacteria at the given concentrations (25 μg/mL for ampicillin and 10 μg/mL for cephriaxim). Overall, fractions from *H. perforatum* show varied antibacterial effects depending on the fraction and concentration, with some fractions demonstrating significant inhibition against both bacteria. In contrast, antibiotics like ampicillin and cephriaxim show minimal activity, as shown in Figure 3. Studying bacteria like *M. cerebrosus* and *E. corrodens* is crucial because they play a significant role in periodontal disease, which affects the gums and can lead to tooth loss if left untreated. These bacteria are particularly concerning due to their resistance to common antibiotics, making it harder to treat infections effectively. Despite their importance, research on these bacteria remains limited, so understanding their behavior and how to combat them is still a growing field of study. Recent research has focused on exploring alternative treatments, such as the impact of phenolic compounds on bacterial growth. These compounds, found in *H. perforatum* , may offer a promising solution to inhibit the development of these bacteria and provide a more effective way to manage gum disease.

**TABLE 3:** Antimicrobial activity of fraction I–IV against *Eikenella corrodens and Morococcus cerebrosus*.

Fractions	Concentration μg/mL	Zone of inhibition (mm)	
		*Eikenella corrodens*	*Morococcus cerebrosus*
I	25	20	20
	50	25	25
	100	22	22
	200	20	20
II	25	0	0
	50	0	16
	100	19	15
	200	20	21
III	25	19	19
	50	0	0
	100	18	18
	200	18	18
IV	25	13	0
	50	18	15
	100	23	0
	200	15	22
Ampicillin	25	0	12
Cephriaxim	10	0	13

Bacterial resistance to antibiotic monotherapy or multiple antibiotic therapy is a spiraling problem because a growing number of infections are becoming harder to treat with current antibiotic treatments, and therefore, serious or even lethal health risks are raised. Hence, there is a critical need for new antimicrobial compounds to fight drug‐resistant microorganisms. Plant‐derived products are a great alternative for discovering new biologically active compounds against hospital‐acquired infections or community‐acquired pathogens. Among them, phenolic compounds are the most significant group. Studying phenolic compounds against antimicrobial resistance is challenging, and several studies confirm their bioefficacy against a vast range of Gram‐positive and Gram‐negative bacteria. *H. perforatum* was tested for its antibacterial activity against four microorganisms: *Escherichia coli* , *S. enteritidis* , *Staphylococcus aureus* , and *Enterococcus faecalis* . It was the most potent against both *S. aureus* and *E. faecalis* . Therefore, the antibacterial activity of the extract can be mainly attributed to its TPC (Kakouri et al. 2023; Alaboo and Mohammed 2023). Although *H. perforatum* extracts have been frequently used as antibacterial agents against many pathogenic bacteria, studies on *M. cerebrosus* and *E. corrodens* are lacking. The antimicrobial activity of St. John's Wort methanol extract at different concentrations was investigated using the disc diffusion method against six strains, including Gram‐negative and Gram‐positive bacteria. The results revealed that the methanol extract showed the highest antimicrobial activity at a concentration of 100 mL‐1 against Gram‐negative bacteria *P. aeruginosa* , followed by Gram‐positive bacteria *S. aureus* (Gül et al. 2021).

Plant phenolic compounds can act against bacterial cells through several mechanisms, considering their antibacterial activity. These include interaction with proteins and bacterial cell walls, alteration of cytoplasmic functions and membrane permeability, inhibition of energy metabolism and DNA damage, or inhibition of nucleic acid synthesis by bacterial cells. It is crucial to understand that the mechanism of inhibition is not a one‐size‐fits‐all but somewhat varies depending on the structure of the phenolic compounds and the bacterial species (Tusevski et al. 2017).

The results indicate that the phenolic compounds extracted from *H. perforatum* have high antimicrobial activity against all the tested microorganisms, such as *S. aureus* , *E. coli* , and *Bacillus subtilis* ‐type organisms (Çelen et al. 2008). Ali et al. (2011) studied the effect of isolated phenolic fractions from *H. perforatum* on several bacteria: *E. coli* , *S. aureus* , and *Pseudomonas aeruginosa* . The phenolic fractions showed some antibacterial activity against all the tested microorganisms, and all the bacteria in the study were sensitive to the phenolic fractions.

### 3.3 Antimicrobial Activities of *H. perforatum* Fractions on Biofilm Formation

Table 4 presents the inhibitory effects of phenolic compounds isolated from *H. perforatum* on the growth and biofilm production of *E. corrodens* and *M. cerebrosus* . The concentrations of the phenolic fractions range from 25 μg/mL to 200 μg/mL, and the data show variation in the impact on both bacterial species. For *E. corrodens* , fraction I at 50 μg/mL showed the most significant inhibition (0.7355), while at higher concentrations (100 μg/mL and 200 μg/mL), the effect slightly decreased. The other fractions (II, III, and IV) exhibited varied results, with fraction II at 200 μg/mL yielding the highest inhibition (0.8659). However, this was more effective than fractions III and IV at similar concentrations. On the other hand, *M. cerebrosus* showed a different response; fraction II at 50 μg/mL had the lowest growth (0.5147), while fraction I at 50 μg/mL produced the second‐lowest growth (0.5093). The highest biofilm inhibition for *M. cerebrosus* was observed with a fraction II at 200 μg/mL (0.4824). Comparing the two bacteria, *E. corrodens* generally had lower inhibition values across all concentrations, with the highest biofilm production in the control group. At the same time, *M. cerebrosus* demonstrated slightly higher susceptibility to the phenolic fractions. The control group, with a bacterial density of 1.5 × 10^8^ CFU/mL, demonstrates susceptibility with respective inhibition zones of 0.8606 for *E. corrodens* and 0.8460 for *M. cerebrosus* . Ampicillin, a standard antibiotic, shows consistent inhibitory effects across concentrations, achieving notable inhibition against both bacteria at 25 μg/mL (0.8502 and 0.6312) and 50 μg/mL (0.8422 and 0.7342). Overall, the phenolic compounds isolated from *H. perforatum* exhibit various antibacterial and biofilm‐inhibitory effects, with the most prominent results in fraction II, particularly for *M. cerebrosus* .

**TABLE 4:** Effect of fractions I–IV against *Eikenella corrodens* and *Morococcus cerebrosus* and biofilm formation.

Fractions	Concentration μg/mL	*Eikenella corrodens*	*Morococcus cerebrosus*
I	25	0.6705	0.6385
	50	0.7355	0.5093
	100	0.6607	0.6072
	200	0.5161	0.5303
II	25	0.5779	0.6024
	50	0,5147	0.6384
	100	0.5633	0.6514
	200	0.8659	0.4824
III	25	0.5729	0.6301
	50	0.5532	0.6780
	100	0.6802	0.6609
	200	0.5430	0.5757
IV	25	0.6977	0.5507
	50	0.7303	0.5001
	100	0.5645	0.6451
	200	0.6670	0.5910
Control	1.5 × 10^8^ CFU/mL	0.8606	0.8460
Ampicillin	25	0.8502	0.6312
	50	0.8422	0.7342
	100	0.7360	0.6400
	200	0.7302	0.5432

*Eikenella corrodens* and *Morococcus cerbrosus*, as Gram‐negative bacteria, present formidable challenges due to their complex cell wall structure. This structure, which includes an outer membrane that acts as a robust barrier against antibiotics, poses a significant hurdle in the fight against antibiotic resistance. The low permeability of this outer membrane, combined with active efflux pumps, severely impedes the entry and retention of antimicrobial agents, leading to the alarming rise of multidrug‐resistant strains among Gram‐negative pathogens (Bisht et al. 2024; Zhang 2022). In this study, it was also shown that *E. corrodens* and *Morococcus cerbrosus* have strong biofilm‐forming capacities. The formation of biofilms by these bacteria further complicates the clinical scenario, as it is associated with persistent infections and antibiotic resistance. The prolific biofilm production by various Gram‐negative species underscores the urgent need for research and intervention in this area (Pawar et al. 2024; Shrestha et al. 2023).

Over the past few years, there has been a notable surge in research focusing on biofilms in the context of periodontal diseases (Colombo and Tanner 2019). *Eikenella corrodens* , a periodontal pathogen, plays a vital role in biofilm formation, which is crucial for its pathogenicity. Microcolonies are formed after 24 h (Azakami et al. 2006). In addition, genetic recombination at the pilin gene locus promotes several pathogenic factors, including biofilm formation, in certain *E. corrodens* strains, suggesting a genetic basis for its virulence (Mansur et al. 2017). The ability of *E. corrodens* to form biofilms is not only relevant to periodontal diseases but also to conditions such as infective endocarditis, where it is part of the HACEK group of bacteria, known for their challenging diagnosis due to their fastidious growth requirements (Cardoso et al. 2005).

There is a significant lack of research on *Morococcus cerbrousus*. However, it is known that this bacterium is capable of forming biofilms. The importance of this knowledge is underscored by the central role that periodontal diseases play in the pathogenesis of many oral diseases and infections. Finding effective alternatives is more pressing than ever because of the difficulty in eliminating these biofilms due to their resistance to antibiotics and host defense mechanisms (Nezhad et al. 2017).

Another study has shown promising results regarding a potential alternative treatment for periodontitis. The study suggests a mixed hydroalcoholic extract of *H. perforatum* could be an effective solution. This extract has demonstrated significant potential in eradicating bacteria in oral biofilms. The study found that at a concentration of 32 mg/mL, *H. perforatum* extract eliminated biofilms in the oral environment, indicating its potential to effectively treat oral diseases associated with biofilms (Vollmer et al. 2019).

Furthermore, the antimicrobial properties of the extracts of *H. perforatum* against bacteria and the formation of biofilm in the mouth showed that the aqueous extract of this plant had a strong antibacterial result in inhibiting the growth of *Streptococcus sobrinus* and *Lactobacillus plantarum* and had a moderate antibacterial effect in inhibiting the growth of *S. mutans* and *E. faecalis* (Süntar et al. 2016). Similarly, it was shown that *H. perforatum* oil was effective against bacteria involved in periodontitis, such as *E. faecalis* , *S. aureus* , *S. mutans* , *E. coli* , and *P. gingivalis*. Regarding oral diseases, the extracts of *H. perforatum* had anti‐biofilm effects that can be used as a natural antibacterial agent in oral health products. The chemical compounds' type, nature, and concentration differ between the aqueous extract and the oil. In addition, plants grown in different areas show different specific chemicals. Furthermore, the polarity of the solvent (methanol, ethanol, acetone, water, etc.) used in the extraction affects its composition (Alahmad et al. 2022; Bagheri et al. 2022).

### 3.4 Antioxidant Activity of *H. perforatum* Fractions

**FIGURE 4:**
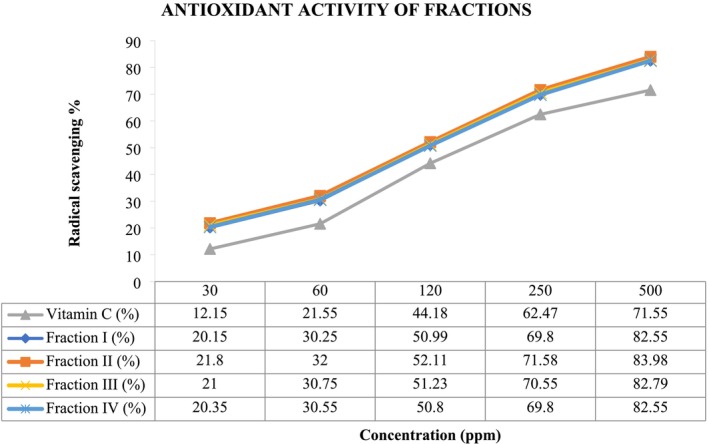
Antioxidant and free radical scavenging activity of isolated fractions.

Figure 4 shows the antioxidant activity (AA%) of various *H. perforatum* fractions, which are known to contain phenolic compounds, measured using the DPPH free radical method. Vitamin C (Vit C) is included as a comparison, and its antioxidant activity increases steadily from 12.15% at 30 ppm to 71.55% at 500 ppm. All four fractions—Fraction I, II, III, and IV exhibited more potent antioxidant activity than vitamin C at lower concentrations, particularly Fraction II, which demonstrated the highest activity, reaching 83.98% at 500 ppm. Fraction III and Fraction I followed closely, showing 82.79% and 82.55% at 500 ppm, respectively. Fraction IV had slightly lower activity than the others but reached 82.55% at the highest concentration. The data underscore the importance of phenolic compounds in *H. perforatum* , as they are known for their antioxidant properties. Phenolics act by neutralizing free radicals, which helps prevent oxidative stress and cell damage. The consistent antioxidant strength of the fractions, especially Fraction II, suggests that these phenolic compounds play a crucial role in the plant's ability to scavenge free radicals, potentially offering therapeutic benefits similar to or surpassing vitamin C at higher concentrations.

*Hypericum perforatum* extracts, containing several classes of plant phenolics with documented biological activity, offer a promising avenue for antioxidant research. The antioxidant flavonoids (mostly quercetin and kaempferol glycosides and aglycones, as well as bioflavonoids) and phenolic acids (mostly isomeric caffeoylquinic acids) in these extracts are a rich source of potential applications. The well‐known antioxidant activity of *H. perforatum* extracts is high due to the high content of phenolic compounds. All fractions extracted from *H. perforatum* were subjected to antioxidant activity assays. They all demonstrated the ability to scavenge DPPH radicals; the observed superoxide anion scavenging further supports the antioxidant properties of these fractions, with IC50 values in the range of 1.86–32.4 μg/mL (Orčić et al. 2011). The procedure described in this paper, enabling the isolation of extract fractions enriched in different groups of phenolics, has opened up new possibilities for understanding the correlation between the extract composition and activity and identifying structural features crucial for scavenging and antioxidant properties. Moreover, anticipating the synergistic action of specific compounds adds a new dimension to our understanding and inspires further research.

Antioxidant properties of secondary metabolites in plants are essential for pharmacokinetics. The antioxidant activity of *H. perforatum* was high in the determination of reducing power activity. DPPH• free radical scavenging activity after the antioxidant compound reacts with DPPH; it is made by measuring the change in color of the DPPH radical at 517 nm (Güzel et al. 2019). Cai et al. (2004) reported on the antioxidant activity of *H. perforatum* , particularly the flower extract, and found that it inhibited free radicals by 32% at an extract concentration of 100 mg/mL. The Trolox equivalent value was 0.83 mM/g DW. Compared to Wojdylo et al. (2007), who reported an antioxidant capacity of *H. perforatum* at 82.3 μM trolox/100 g DW, our results were more promising. Vardapetyan et al. (2014) found the highest radical scavenging capacity in the ethanolic extract of *H. perforatum* leaves. In a study conducted by Rey‐Méndez et al. (2022), the antioxidant activity of three ethanolic extracts obtained from the flowers, leaves, and stems of *H. perforatum* was analyzed using reducing power, total phenolic content, and DPPH scavenging activity assays. The three extracts present high antioxidant capacity, being able to inhibit 90% of the DPPH at the three concentrations tested and presenting low IC50, with mean values of 0.71 ± 0.04, 0.71 ± 0.03, and 0.69 ± 0.004 mg/mL for HPF, HPL, and HPS, respectively. Khodabandeh et al. (2022) reported on the DPPH scavenging activity and TPC of a flower extract of *H. perforatum* obtained with a mixture of ethanol/water for 72 h, with an IC50 value of 0.0281 ± 0.002 mg/mL and TPC of 145.96 ± 9.52 mg GA/g extract.

## 4 Conclusion

This study has revealed the composition, antimicrobial, anti‐biofilm, and antioxidant properties of various *H. perforatum* fractions; it has identified twelve phenolic compounds and demonstrated their activity against antibiotic‐resistant periodontal pathogenic oral bacteria. This study underscores the practical importance of the Soxhlet apparatus for extracting fractions from *H. perforatum* leaves and the effectiveness of different chromatography techniques, such as chromatography column (CC), thin layer chromatography (TLC), and high‐performance liquid chromatography (HPLC). To obtain phenolic compounds in quantity and quality, the bacteria responsible for gum disease, *M. cerebrosus* and *E. corrodens* , contribute to the calcification of dental plaque. Based on the findings of this study, it is recommended that further research be conducted to explore the clinical applicability of *H. perforatum* fractions, particularly in the development of natural therapeutic agents for the treatment and prevention of periodontal diseases. Emphasis should be placed on in vivo studies, formulation development, and safety evaluation to support the integration of these bioactive phenolic compounds into oral healthcare products as potential alternatives or adjuncts to conventional antibiotics.

## Author Contributions

**Siba Mouid Al‐haliem:** methodology, formal analysis, software, writing – original draft. **Muthanna Jasim Mohammed:** investigation, formal analysis, writing – original draft. **Mohammad Ali Hesarinejad:** formal analysis, software, writing – review and editing. **Tarek Gamal Abedelmaksoud:** conceptualization, methodology, formal analysis, data curation, project administration, software, writing – original draft, writing – review and editing.

## Ethics Statement

The authors have nothing to report.

## Consent

All authors have read and agreed to the published version of the manuscript. All authors read and approved the final manuscript.

## Conflicts of Interest

The authors declare no conflicts of interest.

## Data Availability

All data generated or analyzed during this study are included in this published article.
